# Resistome and virulome of high-risk pandemic clones of multidrug-resistant extra-intestinal pathogenic *Escherichia coli* (ExPEC) isolated from tertiary healthcare settings in Uganda

**DOI:** 10.1371/journal.pone.0294424

**Published:** 2023-11-22

**Authors:** Denis K. Byarugaba, Bernard Erima, Godfrey Wokorach, Stephen Alafi, Hannah Kibuuka, Edison Mworozi, Ambrose K. Musinguzi, James Kiyengo, Florence Najjuka, Fred Wabwire-Mangen

**Affiliations:** 1 Makerere University Walter Reed Project, Kampala, Uganda; 2 College of Veterinary Medicine, Makerere University, Kampala, Uganda; 3 Gulu University Multifunctional Research Laboratories, Gulu, Uganda; 4 College of Health Sciences, Makerere University, Kampala, Uganda; 5 Uganda Peoples’ Defence Forces, Ministry of Defence, Kampala, Uganda; Zhejiang University, CHINA

## Abstract

Multi-drug resistant (MDR) globally disseminated extraintestinal pathogenic high-risk *Escherichia coli* (ExPEC) clones are threatening the gains in bacterial disease management. In this study, we evaluated the genomic structure including the resistome and virulome of the *E*. *coli* isolates from extraintestinal infections using whole genome sequencing (WGS). The results highlight that isolates were highly resistant (≥ 90.0%) to commonly used antibiotics (Ampicillin, Trimethoprim-Sulfamethoxazole, Nalidixic acid, and Piperacillin) and were less (<14%) resistant to last resort antibiotics; Imipenem (10.94%) and Meropenem (10.20%). A greater proportion of the *E*. *coli* isolates belonged to phylogroup B2 (30.52%) and phylogroup A (27.37%). The sequence types ST131 of phylogroup B2 (21.05%) and ST648 of phylogroup F (9.3%) were the dominant pandemic high-risk clones identified in addition to the ST1193, ST410, ST69, ST38, ST405, and ST10. Many of the isolates were MDR and most (64.58%) carried the *blaCTX-M-15* gene for extended-spectrum β-lactamases. There was a high correlation between phylogroups and the occurrence of both antimicrobial resistance and virulence genes. The cephalosporin-resistance gene *blaEC-5* was only found in phylogroup B2 while *blaEC-8* and *blaEC-19*, were only found within phylogroup D and phylogroup F respectively. Aminoglycoside gene (*aadA1*) was only associated with phylogroups D and C. The isolates were armed with a broad range of virulence genes including adhesins, toxins, secreted proteases, iron uptake genes, and others. The *yfcv*, *chuA*, and *kpsE* genes preferentially occurred among isolates of phylogroup B2. The study underlines the predominance of MDR internationally disseminated high-risk ExPEC clones with a broad range of virulence genes known to be highly transmissible in healthcare and community settings.

## Introduction

The emergence and global spread of pandemic clones of multi-drug resistant strains of *Enterobacteriaceae* are worrisome [1]. Among them, *Escherichia coli* is the leading cause of both community and healthcare–associated infections. *E*. *coli* pathogenic strains are typically divided into intestinal pathogenic *E*. *coli* (InPEC) and extraintestinal pathogenic *E*. *coli* (ExPEC). ExPEC comprises a highly genetically diverse group with several virulence factors that are responsible for serious extra-intestinal infections including simple urinary tract but also life-threatening bloodstream infections and mortalities [2, 3]. It has a high genomic plasticity that allows it to acquire and share genetic material that enhance its fitness and capabilities to survive in harsh environments. In particular, *E*. *coli* has been demonstrated to harbor several mobile genetic elements (MGEs), such as plasmids, transposons, and integrons, known for facilitating the acquisition and dissemination of resistance genes across strains and different species [1]. These MGEs, plus their high armament of virulence factors are responsible for their successful global transmission and multi-drug resistant strains becoming endemic in most countries. Some of the strains have become resistant to carbapenems and third-generation cephalosporins which belong to the critical category of the World Health Organization’s (WHO) priority list of antibiotics. Infections caused by these strains have very limited treatment options, resulting in extended hospitalizations with high costs and high mortality, especially in resource-limited countries.

Many of the high-risk *E*. *coli* pandemic clones belong to a few of the phylogroups (A, B1, B2, C, D, E, F, and G) and specific multilocus sequence types (MLST). Most studies have reported several pandemic clone sequence types (ST) including ST131, ST648, ST69, ST10, ST405, ST38, ST95, ST73, and ST1193, in health-care associated and community-acquired infections [4–7]. The global distribution of ST131 has been more frequently reported. It is thought to be associated with variants that carry certain resistance plasmids with genes that encode resistance against antibiotics such as extended spectrum beta-lactamase (ESBL), cephalosporins, and fluoroquinolones [8, 9]. The success of this ST131 clone has been largely attributed to the acquisition of many virulence factors and resistance genes which is aggravated by the increased use of antibiotics [10, 11]. The pandemic *E*. *coli* ST131 strains are strongly associated with *bla*CTX-M-15 which is the most predominant ESBL enzyme that hydrolyses beta-lactams. These bacteria and their resistome are frequently shared by animals and humans in the same environment enabling successful interspecies transmission. Pandemic clones such as ST131, ST410, ST648, and ST10 have been reported in domestic animals and birds [12, 13] as they have been in humans further complicating their control.

Despite the availability of sequencing capabilities and national antimicrobial resistance (AMR) surveillance activities, there have been limited detailed characterizations of these pandemic clones in Africa. Such data is critical to estimate the burden and track these strains in the health care and community to improve treatment and management of infections and to institute and evaluate interventions for their containment. Some of the few studies done across sub-Saharan Africa tend to highlight the growing burden of the ESBL and multi-drug resistance (MDR) clonal groups in hospital and community infections especially the ST131 [4–7]. Our study set out to establish the genomic population structure of ExPEC isolates recovered from tertiary healthcare settings in Uganda to identify potential high-risk pandemic clones and their resistome and virulome that may pose a challenge in the management of their infections.

## Materials and methods

### Study area

The samples were collected from patients who received healthcare services from three government hospitals in Uganda. These hospitals were Gulu Regional Referral Hospital, Bombo Hospital, and Bwera General Hospital. The Gulu Regional Referral Hospital is in northern Uganda and has a bed capacity of 370. It receives medical referral cases from Amuru, Gulu, Kitgum, Lamwo, and Pader districts. Bombo Hospital is in the central part of Uganda with a bed capacity of 250. It is designed to provide healthcare services to military personnel, their families and the civilian population around them. The Bwera General Hospital is in Kasese District located in the Western Region of Uganda and has a bed capacity of 200. This hospital borders the Democratic Republic of Congo and receives patients from the Democratic Republic of Congo. All the samples were collected between the year of 2013 to 2020

### Study setting

The samples for bacterial isolation were collected from wounds, urine, pus, and endocervical swabs, and others. The samples were obtained from inpatients, and outpatients. Samples were directly inoculated on MacConkey agar (Oxoid, Remel Inc USA) and incubated at 37°C for 24 hrs. A single colony of lactose-fermenting bacteria were sub-cultured on Eosin methylene blue (EMB) agar (Oxoid, Remel Inc USA) at 37°C for another 24 hrs. Colonies with metallic sheen appearance were picked and presumptively identified as *E*. *coli* based on API-20E kits (bioMérieux—Boston, MA, USA) and later confirmed upon sequence analysis. The isolates were further sub-cultured onto nutrient agar and pure colonies stored in Brain Heart Infusion (BHI) broth (Oxoid, Manchester, UK) containing 50% glycerol at -80°C until further analysis.

### Antimicrobial susceptibility and selection of MDR isolates

The isolates were subjected to antimicrobial susceptibility testing against 17 antimicrobials by disc diffusion assay as previously described [14] on Mueller-Hinton agar (MHA) (Oxoid, Manchester, UK). A suspension of 0.5 McFarland standard turbidity was spread on the surface of MHA plates using a sterile cotton swab. Antibiotic discs with the corresponding strengths indicated in parenthesis: amikacin (AMK30μg), gentamicin (C10μg), ampicillin (AMP10μg), cefotaxime (CTX30μg), amoxicillin-clavulanic acid (AMC20/10μg), ceftazidime (CAZ30μg), ceftriaxone (CRO30μg), cefuroxime (CXM 30μg), trimethoprim-sulfamethoxazole (SXT1.25/23.75μg), chloramphenicol (C30μg), tetracycline (TE30μg), ciprofloxacin (CIP5μg), nalidixic acid (NA30 μg), nitrofurantoin (F300μg), imipenem (IPM10μg), ertapenem (ETP10μg) and meropenem (MEM10μg) were placed onto MHA agar plates and incubated at 37°C for 24 hours. The zone of inhibition was measured to the nearest millimeter and interpreted based on the guidelines of the Clinical and Laboratory Standards Institute (CLSI M100 Ed33) [15] using *E*. *coli* ATCC® 25922 as a control strain. *E*. *coli* that showed resistance to three or more classes of antimicrobial agents were classified as multidrug resistant (MDR) [16] and subjected to whole genome sequencing.

### Whole genome sequencing, assembly, and annotation

Whole genome sequencing was performed as described [17]. In summary, libraries were prepared with Kapa HyperPlus library preparation kits (Roche Diagnostics, Indianapolis, IN, USA). The concentration of the prepared library was determined using the Kapa library quantification kit Illumina/Bio-Rad iCycler (Roche Diagnostics) in a CFX96 real-time cycler (Bio-Rad, Hercules, CA, USA) and sequencing was done on Illumina NextSeq (Illumina, Inc., San Diego, CA) at Walter Reed Army Institute (WRAIR) Multidrug-Resistant Organism Repository and Surveillance Network (MRSN). Btrim was used to remove sequence adapters and regions with low-quality base calls [18]. *De novo* raw reads were assembled using Newbler (v2.9) [19]. Contigs were annotated using DFAST pipeline version 1.2.18 [20]. The sequences were deposited to the NCBI database under BioProject ID PRJNA955428 (https://www.ncbi.nlm.nih.gov/bioproject/955428/).

### Genome sequence analysis

The SNPs calling, filtering, and SNP site validation from the assembled genome sequences (n = 95) was done with CSI Phylogeny [21]. A phylogenetic tree was constructed using the concatenated alignment derived from high-quality SNPs and the tree was viewed and annotated using Interactive Tree of Life (https://itol.embl.de/). The genetic diversity was determined based on phylogroup, Multi Locus Sequence Typing (MLST), Serotypes, and Fimtypes. The phylogroup typing was based on the ClermonTyping scheme and was done according to Beghain and others [22]. Multilocus Sequence Typing of assembled genome sequences was done using MLST v2.0 database [23]. SerotypeFinder v2.0 was used to assign the isolates to their corresponding serotypes [24]. FimTyper v1.0 database curated by Henrik Hasman was used for the classification of *E*. *coli* isolates into different *FimH* types [25]. Characterization of the *E*. *coli* into H30Rx subclones was done based on profiling single nucleotide mutation within peptide antibiotic transporter and putative allantoin permease genes [26]. A combination of ResFinder 4.1, AMRFinder, and CARD databases was used to identify and confirm the presence of acquired antibiotic-resistance genes within the genome of the *E*. *coli* strains [27–29]. The occurrence of different virulence genes within the genome of the *E*. *coli* isolates was determined using VirulenceFinder v2.0 [30]. Isolates with these four virulence genes (*chuA*, *fyuA*, *vat*, and *yfcV*) were assigned as presumptive uropathogenic strains as described [31]. Contigs were assigned as derivatives from plasmids using a combination of mlplasmids v2.1.0, PlasmidFinder v2.1, and NCBI blast [32, 33]. The plasmid replicons for contigs derived from plasmids were determined using PlasmidFinder v2.1. The location of AMR genes within contigs that were confirmed to be derived from plasmids was determined using ResFinder 4.1, AMRFinder, and CARD database. The arrangement of the AMR genes within contigs derived from the plasmid was mapped using clinker software [34]. Default parameters were used for each of the software unless specified otherwise.

### Ethics approval

This work was undertaken under “Protocol RV 309, Antimicrobial Resistance Surveillance in Uganda”, approved by the Makerere University School of Public Health Higher Degrees and Research Ethics Committee (HDREC 087), Uganda National Council of Science and Technology (HS775) and Walter Reed Army Institute of Research IRB (WRAIR #1711).

## Results

### Phenotypic antimicrobial susceptibility of the isolates

**Fig 1:**
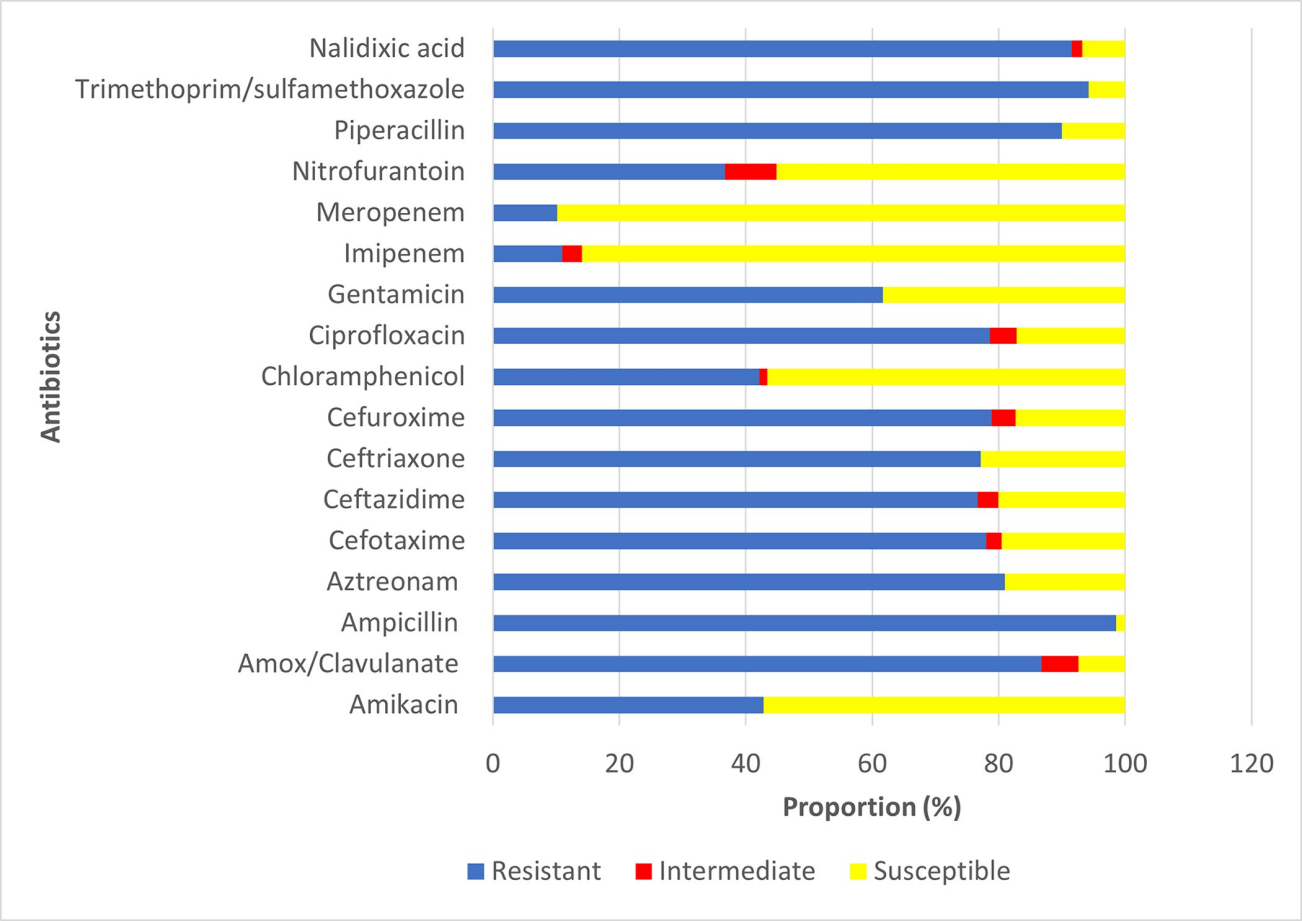
Antimicrobial susceptibility of the isolates.

The 95 isolates were recovered from wounds (n = 4), pus (n = 29), urine (n = 57), endocervical swabs (n = 2), devices (n = 1), and others (n = 2) among patients in the outpatient departments (OPD) and inpatient departments (IPD). The number of isolates tested for each antibiotic varied and the frequency of resistance is summarized in Fig 1. The results highlight that most of the *E*. *coli* isolates were susceptible to imipenem (85.94%) and meropenem (89.80%) antibiotics. However, for the other antibiotics tested, a higher proportion of isolates with resistant traits were observed. For example, the proportion of isolates resistant to ampicillin, trimethoprim-sulfamethoxazole, nalidixic acid, and piperacillin were ≥ 90.0%. Isolates resistant to amox/clavulanate (86.76%) and aztreonam (80.95%) were equally high in proportion. The proportion of isolates resistant to cefotaxime, ceftazidime, ceftriaxone, and cefuroxime was nearly the same and was in the range of 76.67% ^__^78.85%. Overall, a significantly higher proportion of isolates were multi-drug resistant (MDR).

### Phylogroups, serotypes, *fimH* type, and sequence type of the *E*. *coli* isolates

**Fig 2:**
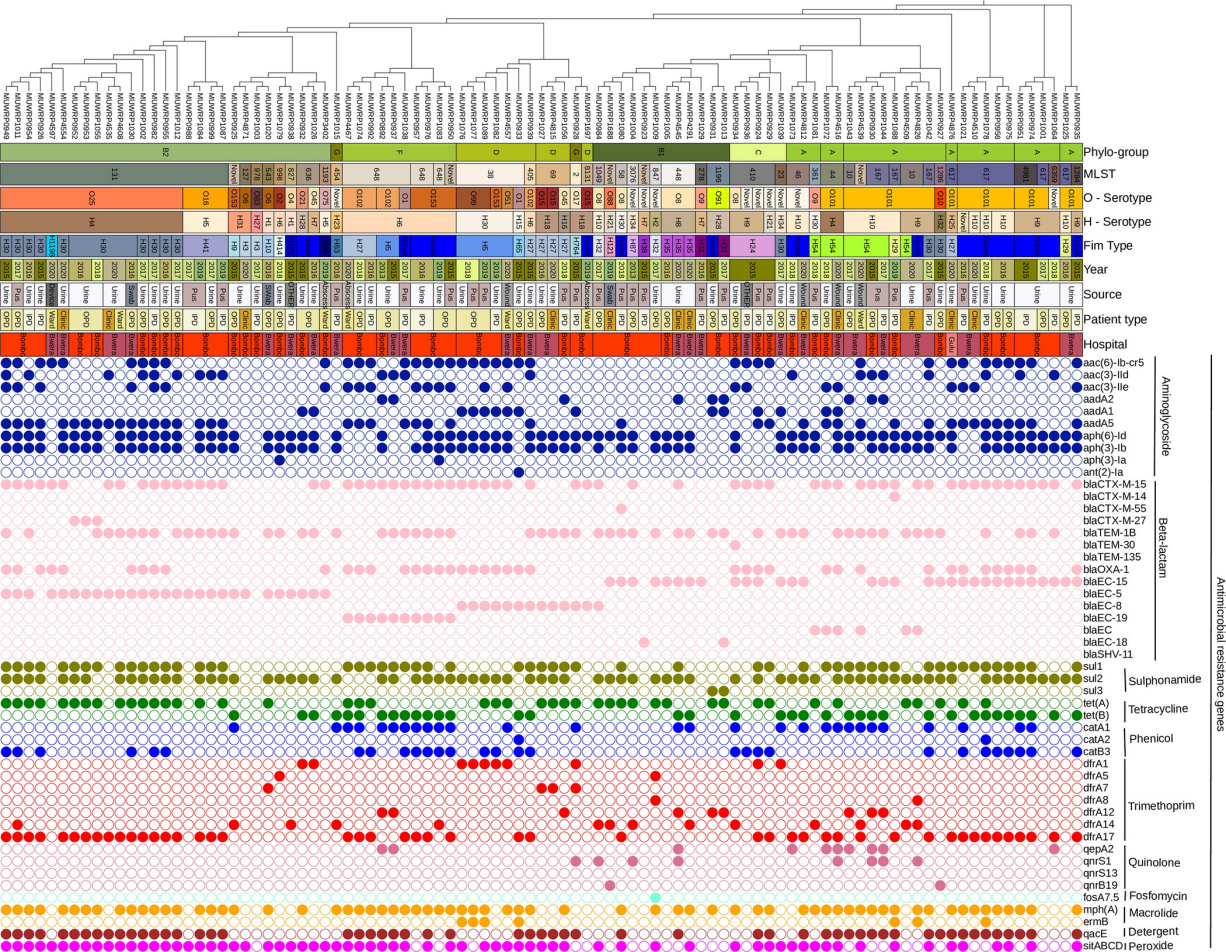
Core genome SNP-based phylogenetic tree of the 95 *E*. *coli* strains analyzed in this study characterized by phylogroup, sequence type (ST), serotype, and fimtype with the corresponding antimicrobial resistance genes (shown in colored circles according to class of antibiotics). The hospital codes (Bwera: Bwera Hospital, Bombo: Bombo Hospital, and Gulu: Gulu Hospital). The figure was produced using the iTOL tool.

The isolates belonged to diverse phylogroups, serotypes and sequence types (Fig 2). Isolates were distributed in seven phylogroups: A, B1, B2, C, D, F, and G. Phylogroup B2 (30.523%) and phylogroup A (27.37%) had the highest frequency of occurrence and occurred in nearly equal proportion between the two hospitals. A marginal difference was observed in the proportion of *E*. *coli* isolates in phylogroup B1 (12.63%), phylogroup D (11.58%), and phylogroup F (10.53%). Phylogroup C (5.26%) and phylogroup G (2.10%) had a marginal proportion of occurrence. Multi-locus sequence typing revealed high genetic diversity among the isolates clustering into 34 different MLST groups. The ST131 (21.05%) and ST648 (9.47%) were the dominant STs and belonged to the globally disseminated pandemic high-risk clones. Other globally disseminated high-risk clones detected were ST1193, ST410, ST69, ST38, ST405, and ST10. Most of the isolates in phylogroup B2 were ST131 clonal group (68.96%) while the other STs were distributed across the different phylogroups. The high-risk clones (ST131, ST648, ST410, and ST69) were distributed in both hospitals whereas all the ST10 isolates were from Bwera General Hospital. *In-silico* serotyping showed the isolates similarly diversified belonging to 24 different O-serotypes with O101(22.11%) and serotype O25 (16.84%) being predominant. A proportion (10.24%) of the isolates could not be typed into a known serotype group (Fig 2). Several H-serotypes were identified including H4 (18.95%), H10 (13.68%), H6 (12.63%), H9 (11.58%), H30 (7.37), H5 (5.26%), H7 (4.21%), and H18 (4.21%) (Fig 2). The different major H-serotypes were observed to occur in both hospitals. However, the frequency of occurrence of H-serotypes follows that of phylogroups and STs. For example, the H4 serotype was distributed only among phylogroups B2 (16.84%) and A (n = 2.1%). As well, the H9 serotype was distributed among phylogroup A (9.47%) and phylogroup C (3.16%). Similarly, the majority of serotype H10 occur within phylogroup A. H6 serotypes were restricted within only phylogroup F and ST648 (Fig 2).

The isolates also had a high diversity of *fimH* types distributed across the different phylogroups and STs with *H*30 clones being the predominant one found mostly within the ST131 (Fig 2). A total of 50% of the *H*30 clones belong to *H30-Rx* groups which originated from an H30-R sub-clone. All the ST131-H30 clones tested were resistant to ciprofloxacin, nalidixic acid, and several beta-lactams. A greater proportion of *H30* isolates were found to carry the *blaCTX-M*-15 gene.

### Detection of AMR genes

**Fig 3:**
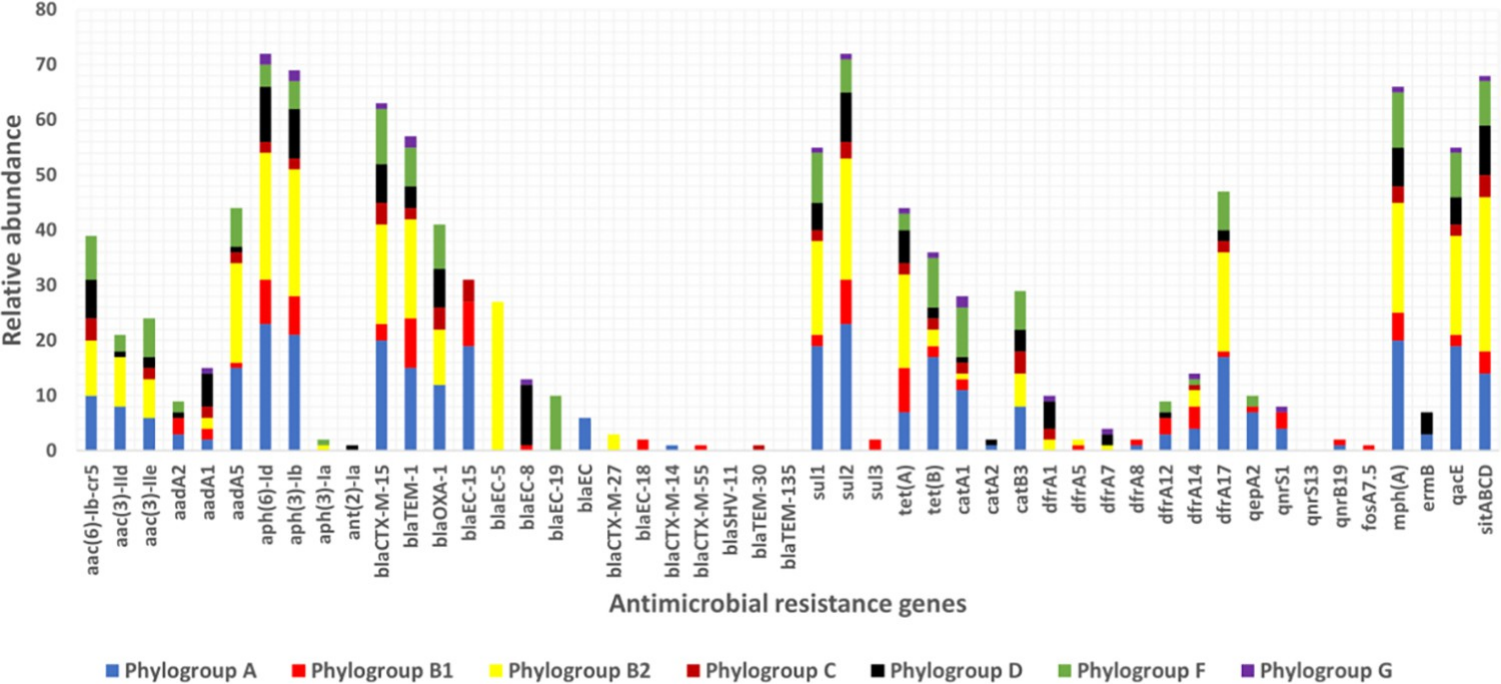
Relative abundance of antimicrobial resistance genes among *E*. *coli* phylogroups.

Several AMR genes for different classes of antibiotics (aminoglycosides, beta-lactams, sulphonamides, tetracyclines, macrolides, and trimethoprim) were detected (Fig 2). Most of the isolates carried multiple resistance genes with a wide distribution for specific genes in the different classes of antibiotics. The proportion of the following aminoglycosides resistance genes *aph (6)-Id* (75.79%), and *aph (3)-Ib* (72.63%) was high among the *E*. *coli* isolates while *sul2* (75.79%) and *sul1* (57.89%) genes were the most frequent sulphonamide resistance genes (Fig 2). A total of 69.47% of isolates were detected with macrolide-resistance gene *mph(A)*. The *blaCTX-M-15* was widely distributed (66.32%) across the different phylogroups and so was *blaTEM-1B* (60.00%) and *blaOXA*-1 (43.16%). The *blaCTX-M-27*, which has been of global concern was detected in three isolates of the ST131-O25 serotype in phylogroup B2. Tetracycline resistance genes *tet(A)* (46.32%) and *tet(B)* (37.89%) were also widely spread among isolates. Antibiotic-resistant genes such as *aph(6)-Id*, *aph(3)-Ib*, *aadA5*, *blaCTX-M-15*, *blaTEM-1*, *tet(A)*, *tet(B)*, *catA1*, *dfrA17*, *mdf(A)*, *sitABCD*, *mph(A)*, and *qacE* were found in all phylogroups (Figs 2 and 3). However, an apparent association of some resistance genes with some *E*. *coli* phylogroups was observed. For example, the *blaEC-8* gene majorly occurred among isolates of phylogroup D whereas the *blaEC-19* gene was detected only within phylogroup F, and the *blaCTX-M-14* gene was restricted to phylogroup A (Figs 2 and 3). Fosfomycin‐resistance gene *fosA*7.5 was the only gene detected in one isolate for fosfomycin resistance and only detected in a novel serotype in ST846 belonging to phylogroup B1. The *qnrS1*-gene occurs in (8.42%) of isolates derived from pus and urine samples. The isolates carrying *qnrS1*-gene were of phylogroups (B1 and A) and serotypes (O101 and O8). Similarly, the two isolates that had the *qnrB19* gene were of phylogroups (B1 and A) but of serotypes (O10 and O88). Among the high-risk clones, ST1193 and ST405 isolates were found to carry *blaCTX-M-15* and *blaOXA-1* besides *aac(6)-Ib-cr*5 and *aac(3)-IIe* genes. The *blaEC*-5 gene preferentially occurs among the high-risk clone ST131. Also, the preferential occurrence of the *blaEC*-19 gene was observed among the ST648 clone. The *catB3* and *aac (6)-Ib-cr5* genes were most detected among the ST410 clone. The following genes *blaEC-8*, *tet(A)*, *blaTEM-1B*, *sul1*, *aph (3)-Ib*, *sul2*, and *aph (6)-Id* were present in all the ST69 high-risk clones. A higher proportion of (*blaEC-8*, *aadA1*, *blaOXA-1*, *blaCTX-M-15*, *aph (3)-Ib*, *sul2*, and *aph(6)-Id)* was observed among high risk clone ST38. Chromosomal point mutations within the *gyrA* gene responsible for ciprofloxacin resistance phenotype were detected in 82.1% of the isolates. The observed change in nucleotide was from TCG to “TTG/GTG” in the isolates detected with *gyrA* mutation (S1 File). Similarly, a mutation within the *parC* gene occurred in 73.68% of the isolates. The mutation within the *parC* gene led to the changes in nucleotide from codon AGC to ATC or AGC to ATT which resulted in a change in amino acid from serine (S) to isoleucine (I) (S1 File). A total of 72.63% of isolates had a mutation in both *parC* and *gyrA* genes that is known to result in ciprofloxacin resistance phenotypes.

### Antibiotic-resistance phenotypes and genotypes among high-risk sequence types

The highest proportion of high-risk clone, ST131 isolates were resistant to quinolones and extended spectrum beta lactam antimicrobials. However, the majority of the ST131 tested were susceptible to chloramphenicol and amikacin. A similar resistant pattern to ciprofloxacin was observed in all the isolates of high-risk group ST648 and ST410 that were tested, while, all the isolates of the high-risk clone ST69 tested were susceptible to ciprofloxacin antimicrobials but were all resistant to trimethoprim-sulfamethoxazole and ampicillin. There was an observed relationship between some antimicrobial-resistance genes and the antimicrobial resistance phenotypes among the high-risk clone ST131. Trimethoprim resistance phenotype was particularly high among ST131 isolates (81.25%) with the *dfrA17* gene (S1 File). A similar pattern of antibiotic resistance phenotypes was observed with beta-lactam antibiotics tested against the ST131 clone. For example, a high level of resistant phenotypes to ampicillin (86.67%), aztreonam (85.7%), cefotaxime (100%), ceftazidime (78.57%), ceftriaxone (83.33%), and cefuroxime (90.91%) antibiotics were observed predominantly among the ST131 isolates with *blaCTX-M-15* gene (S1 File). Moreover, a greater proportion of ST131 isolates with *blaEC-5* gene were resistant to ampicillin (93.33%), aztreonam (85.71%), cefotaxime (100%), ceftazidime (85.71%), ceftriaxone (88.89%), and cefuroxime (81.81%) antibiotics (S1 File).

### Distribution of plasmids and resistance determinants

**Fig 4:**
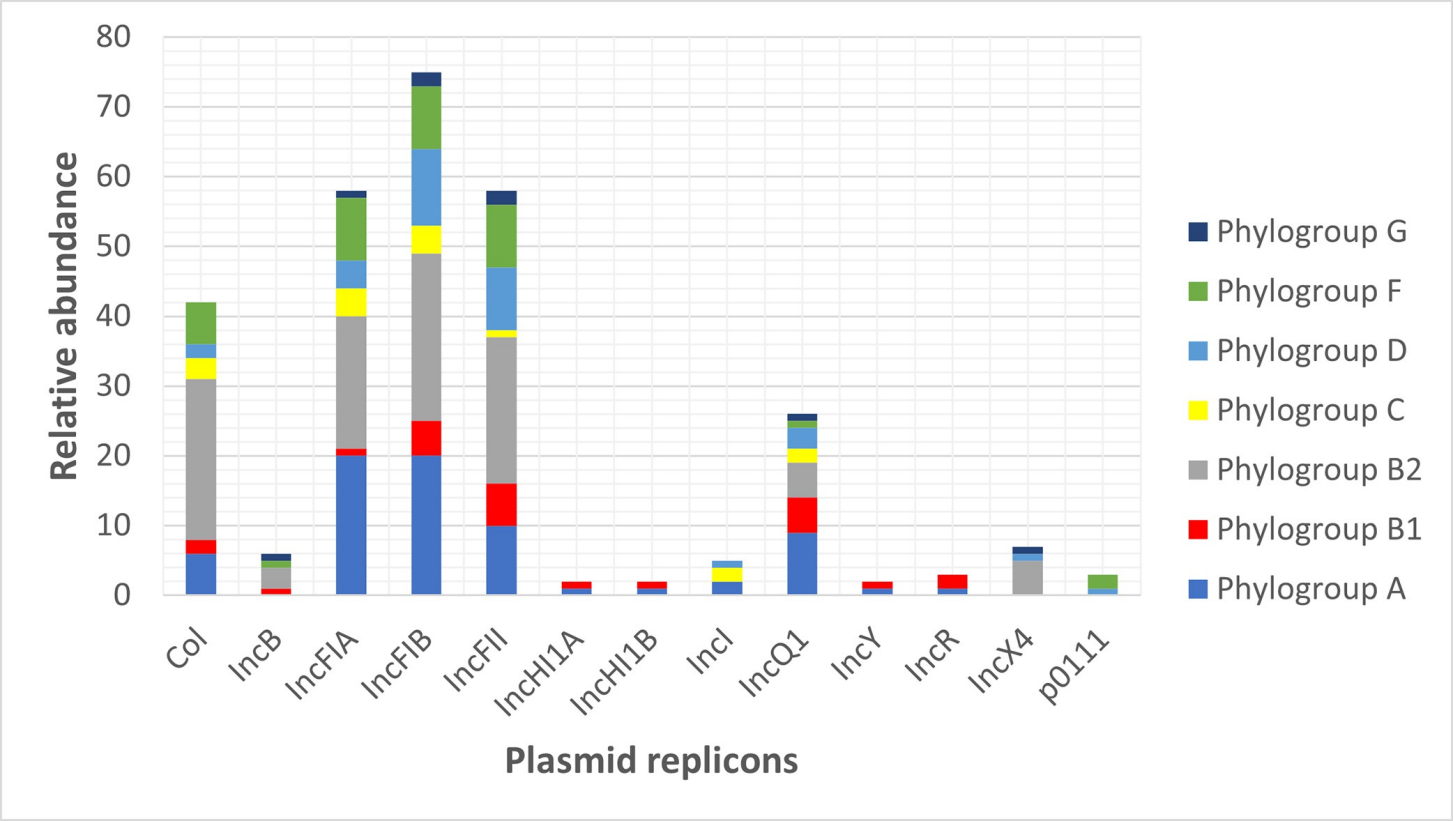
Relative abundance of plasmid replicons among *E*. *coli* phylogroups.

Several classes of plasmid replicons were identified on some of the contigs derived from the plasmids (Fig 4). The most frequent plasmid replicons detected were *IncFIB* (n = 72), *IncFIA* (n = 57), *IncFII* (n = 57), *IncQ1* (n = 26), *Col156* (n = 20), and *Col (BS512)* (n = 11) (Fig 4). Plasmid replicons *IncFIA*, *IncFIB*, *IncFII*, and *IncQ1* were found in all phylogroups. However, some replicons were only present in a few phylogroups including *IncHI1A* and *IncHI1B* which were detected in only one isolate of phylogroup A and phylogroup B1 respectively. Only one isolate of phylogroup D and two isolates of phylogroup F were detected with plasmid replicon *p0111*. Also, two isolates each from phylogroups A and C, and one isolate of phylogroup D had plasmid replicon *IncI*. None of the two isolates of phylogroup G had plasmid replicons *Col*, *IncHI1A*, *IncHI1B*, *IncI*, *IncY*, *IncR*, and *p0111* (Fig 4). We observed that contigs/fragments having some plasmid replicons were associated with the occurrence of certain *AMR* genes. For example, aminoglycoside resistance genes (*aph (3’’)-Ib* and *aph (6)-Id*) and sulphonamides resistance gene (*sul2*) were located within contig carrying plasmid replicon *IncQ1*. *blaTEM-1B* mainly occurred within the contigs having plasmid replicon *IncFII*.

**Fig 5:**
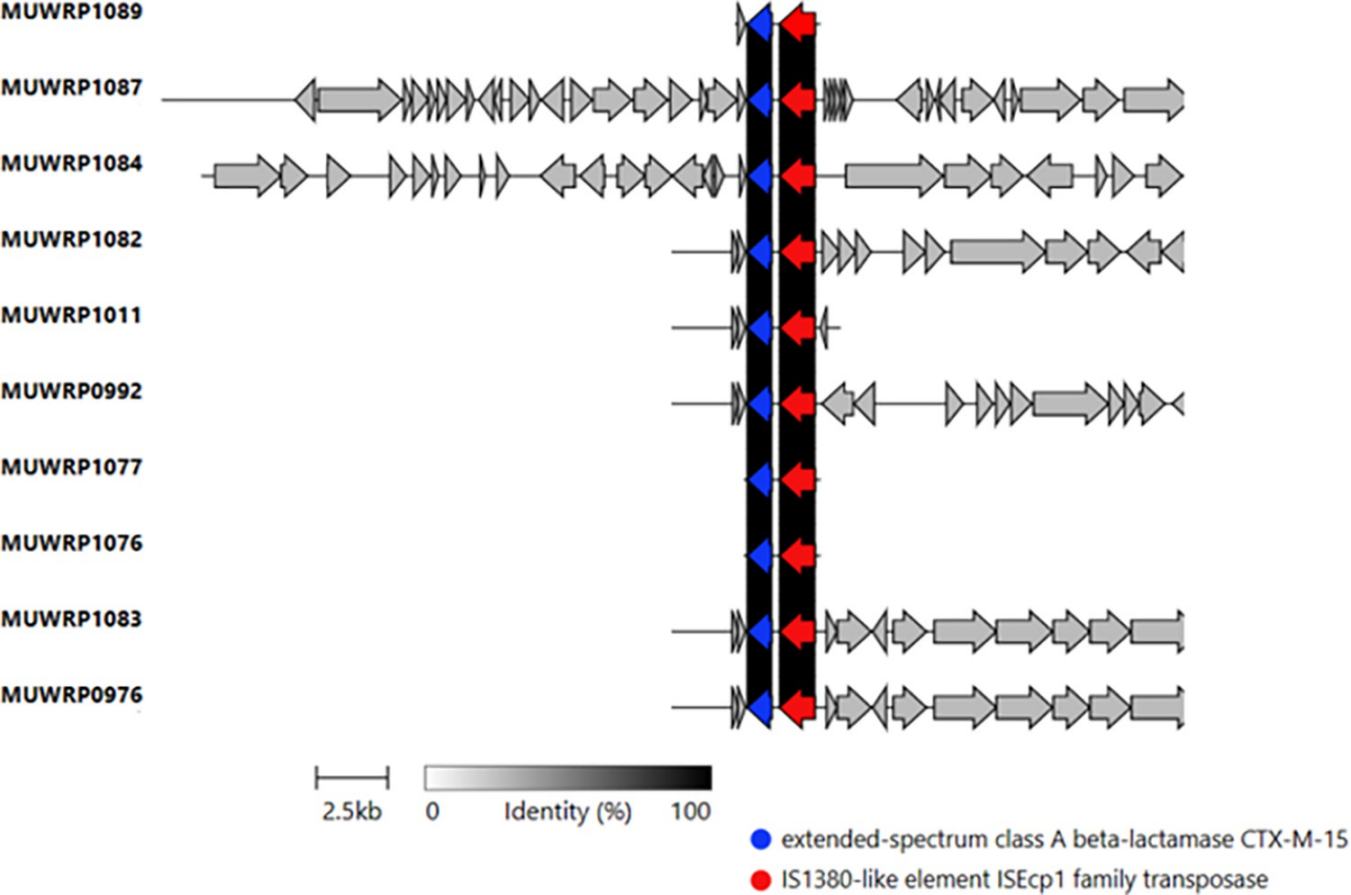
Gene arrangement showing a close association between antimicrobial resistance gene *blaCTX-M-15* and mobile genetic element *ISEcp1*.

Some plasmid contigs or fragments-derived plasmids carry multiple antibiotic resistance genes. For example, the aminoglycoside gene (*aac(6’)-Ib-cr*) was often found to occur in the same contig as the amphenicol resistance gene (*catB3*) and beta-lactam resistance gene (*blaOXA-1*). A total of 35.41% of isolates had plasmid contigs having both (*aac(6’)-Ib-cr*), (*catB3*), and (*blaOXA-1*). As well, the aminoglycoside resistance gene (*aadA5*), sulphonamide-resistant gene (*sul1*), and macrolide resistance gene *mph(A)* were carried together in the same plasmid contigs. The *blaCTX-M-15* genes were located downstream of the mobile genetic element *ISEcp1* (Fig 5).

### Characteristics and distribution of virulence genes

**Fig 6:**
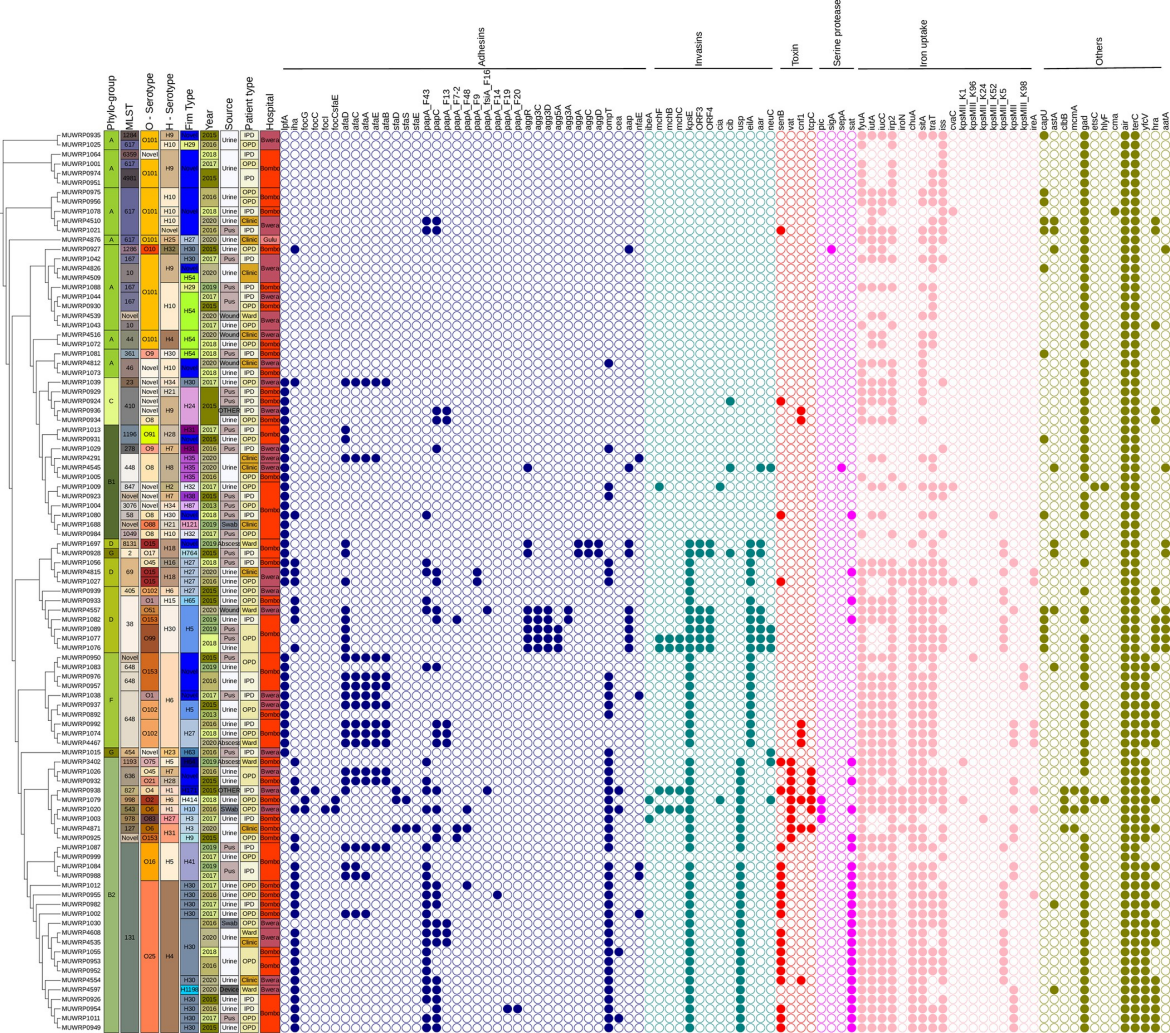
Core genome SNP-based phylogenetic tree of the 95 *E*. *coli* strains analyzed in this study characterized by phylogroup, sequence type (ST), serotype, and fimtype with the corresponding virulence genes (shown in colored circles according to the virulence gene group). The hospital codes (Bwera: Bwera General Hospital, Bombo: Bombo Hospital, and Gulu: Gulu Regional Referral Hospital). The figure was produced using the iTOL tool.

Numerous virulence genes were detected (Fig 6 and S1 Fig). All the isolates had Enteroaggregative immunoglobulin repeat protein (*air)* and Tellurium ion resistance protein (*terC)*. Other frequently detected virulence genes were glutamate decarboxylase (*gad)* (83.33%), siderophore receptor (*fyuA)* (75%), high molecular weight protein 2 non-ribosomal peptide synthetase (*irp2)* (75%), iron transport protein (*sitA)* (70.83%), and Ferric aerobactin receptor (*iutA)* (66.62%) (Fig 6 and S1 Fig). Other virulence genes that were detected at moderate proportions were *kpsE*, *chuA*, *iss*, and *ompT*. The pattern of occurrence of other virulence genes was determined by the phylogroups of the *E*. *coli* isolates except for *air*, *terC*, and *gad* genes that occur at high frequency in all phylogroups. All the isolates of phylogroups B1, F, and C had virulence gene *lpfA* whereas none of the isolates of phylogroups (B2 and A) had the gene (Fig 6 and S1 Fig). The *yfcv* gene was detected only among phylogroups B2 and F. Similarly, the *chuA* and *kpsE* virulence genes were detected only among phylogroups (B2, D, and F) with a higher proportion of occurrence (Fig 6 and S1 Fig). Generally, Phylogroup B1 and A lacked most of the virulence genes that tend to occur in phylogroup B2. A total of 13.85% of the isolates were detected with all four virulence genes (*chuA*, *fyuA*, *vat*, and *yfcV*) that define uropathogenic strains of *E*. *coli*. All the presumptive uropathogenic strains detected were of phylogroup B2 and were randomly distributed among seven different sequence types (ST127, ST543, ST636, ST827, ST978, ST998, ST1193) and the two hospitals.

## Discussion

Our study identified highly diverse virulence and multidrug resistance genes among ExPEC with predominance of the globally disseminated high-risk pandemic clones. These clones have been reported with high frequency and pose a threat to the management of both community and hospital-acquired infections because of their high transmission in both settings [35–37]. *Escherichia coli* is a normal inhabitant of the gastro-intestinal system but can cause serious clinical conditions including bloodstream infections, urinary tract infections, meningitis, and diarrhea, some of which have very high mortality. The high genetic diversity and increasing resistance of *E*. *coli* has been reported in many parts of Africa [38]. Also *E*. *coli* is reported as being responsible for the biggest proportion of the burden of AMR globally [39].

Our study revealed the dominance of the phylogroups B1, A, and B2 with most of the isolates belonging to one of the pandemic high-risk ST131 clones. While several ExPEC lineages have been reported to be responsible for human extraintestinal infections, most reports suggest that specific lineages are responsible for the major burden of these infections despite their STs being genetically diverse [2]. Indeed, we reported a high genetic diversity with 34 STs, 24 different serotypes, and several fim types further confirming the differences in the genetic background that were also found with highly divergent virulence, and resistance gene carriage thought to be mediated by persistence and adaptation in the intestinal tract. Our findings are consistent with other reports in Uganda [7, 40] and in other African countries [41]. The success of some of the most successful pandemic clones is attributed to the broad armament with virulence genes but also their resistance to the barrage of antimicrobials used commonly in healthcare and community settings. Our study revealed the occurrence of several of these international high-risk pandemic clones including ST131, ST648, ST38, ST405, ST1193, ST410, and ST10. These strains have been reported globally causing not only community-acquired infections but also healthcare-associated infections, worldwide.

ST131 was the most dominant clonal group and was isolated from urine, pus, and abscesses in both geographical areas highlighting they are widely spread within Uganda. ST131 is known to be associated with serious or fatal extra-intestinal infections [42, 43] which is of clinical and public health importance. The ST131 clone is notably responsible for the global spread of AMR in *E*. *coli*, especially against high-priority antibiotics such as fluoroquinolones and extended-spectrum cephalosporins (ESCs). ST131 emerged around the year 2000 and rapidly spread across the world and became the predominant ExPEC clone throughout the world [44]. It has been reported within African countries such as Malawi, Nigeria, Congo, Rwanda, and many other countries [3–5, 45]. We identified two O serotypes in our study O25-ST131-H30R and O16-ST131-H41R similar to what is commonly reported elsewhere [46–48]. The O25-ST131-H30 identified in this study has been more predominant in many similar studies and has emerged as the major clonal group in Uganda [49]. All the O25-ST131 strains were of the fimbrial adhesin gene *fim*30 type except one that was *fimH1190*. The ST131-*H*30 is extensively resistant and has been globally and epidemiologically a successful clonal subset, designated so because it contains allele 30 of the type-1 fimbriae adhesin gene *fimH* [9, 26]. Two dominant antimicrobial resistance sub lineages have been identified within ST131-H30/clade C: H30R which is characterized by resistance to fluoroquinolones, and H30Rx (or clade C2), which is characterized by resistance to fluoroquinolones as well as production of a *CTX-M-15* type extended-spectrum beta-lactamase (ESBL) that confers resistance to extended-spectrum cephalosporins [50–52]. Half of the ST131 isolates in our study were ST131 C1/*H*30R while the other half were ST131 C2/*H*30Rx and these sub clonal groups produced CTX-M-15 consistent with reports from other studies [53]. Our study detected ST131 with the *blaCTX-M-27* gene which used to occur in Japan and is getting more frequently reported globally [54]. The current shift and spread of the *blaCTX-M-27 E*. *coli* and its pandemic potential are still largely not well understood. The other common MLST group in our study was ST-648 clonal group which has been reported to have the potential of carrying more non-β-lactam antimicrobial drug resistance genes [55]. However, besides these, there were several other sequence types among different phylogroups with highly variable serotypes and *fimH* types demonstrating the extensive genetic diversity of EXPEC in our study. This observation is consistent with other studies in Uganda and elsewhere [3–5, 37].

Most of the STs are particularly known for their association with extensive resistance to several antibiotics. The *E*. *coli* isolates from both hospitals carried multiple resistance genes. The most common ones observed were against the commonly used antimicrobials such as penicillins, tetracyclines, sulphonamides/trimethoprim which is consistent with the phenotypic resistance observed and commonly reported in many countries including Uganda [56–58]. Of major concern, is the finding of resistance genes to third-generation cephalosporin-resistant that belong to the WHO priority list of critical antimicrobials. Most of the isolates in our study carried resistance genes to extended beta lactams and third and fourth generation cephalosporins including *blaCTX-M-15*, while a few *blaCTX-M-14*, *blaCTX-M-55* were reported and have also been observed in other studies in Uganda [59]. The *blaCTX-M-15* and *blaOXA-1* genes were distributed among the different MLST groups including the pandemic clones of ST-648, ST-617, and ST-131 which also carried several antimicrobial resistance genes. In addition, the *CTX-M-27* was reported in only 3 isolates. This resistance gene was first reported in Japan and has been increasing and causing serious concerns because of its presence in clonal groups such as ST10, ST69, and ST131 [60–62]. In the current study, this *blaCTXM-27* was only found within the O25-ST131. Furthermore, we identified a f*osA*7.5 resistance gene responsible for resistance against fosfomycin in a lone ST847 with a novel serotype and *fimH32* that was the only isolate among all isolates. Fosfomycin is often the last resort antibiotic used against MDR *E*. *coli* strains and increasing resistance has been reported globally mainly due to *fosA*3 gene [63–65]. A high prevalence of *fosA*-7.5 gene was reported on animal farms in China which may confirm that food animals may serve as a potential reservoir for the resistance genes especially due to the frequent association with mobile elements, that would accelerate the transmission of *fosA*-like gene in *E*. *coli* strains [66]. We also identified *blaEC* gene that codes for cephalosporin resistance in specific phylogroups and MLSTs specifically *blaEC-5*, *blaEC-19*, and *blaEC-8* in phylogroup B2, F and D respectively. The management and treatment of life-threatening infections caused by multidrug resistance (MDR) bacteria are challenged by an increase in resistance to third and fourth generation cephalosporins (broad-spectrum β-lactam antimicrobial agents) which are among the list of antibiotics categorized as critically important by world health organization. While most of the high-risk clones also showed resistance to fluoroquinolones, we observed only a few isolates 8/95 and 2/95 with *Qnr* protective proteins *qnrS1* and *qnrB*19 respectively shown to be associated with low-level resistance. The carriage of the resistance genes on mobile genetic elements (MGEs) such as plasmids has been reported in several studies and is highly responsible for the successful transmission of AMR. We profiled contigs to trace resistance gene sources and observed that couples of AMR genes were derived from plasmids. It was observed that all contigs with plasmids replicon *IncFIB* had antibiotic resistance gene *blaTEM-1B* while contigs with *IncR* plasmid replicons were found in association with many resistant determinants such as *qacL*, *aadA1*, *sul3*, *dfrA12*, *aadA2*, and *cmlA1*.

The success of these MDR strains is partly due to the simultaneous possession of a wide range of virulence factors besides the resistance to antimicrobial agents. *E*. *coli* has a variety of virulent factors including toxins, iron/heme-acquisition systems, adhesins, and iron ion transport. Fimbriae is critical for successful attachment (adhesion) to surfaces (epithelial cells) of intestines, kidneys, or lower urinary tracts, in order to establish extraintestinal infections. Our study revealed a diverse collection of virulence genes that are associated with *E*. *coli* isolates. Most of the isolates had the enteroaggregative immunoglobulin repeat protein (*air*) virulence gene implicated in the promotion of *E*. *coli* aggregation and adherence as well as tellurium ion resistance protein (*terC)*, glutamate decarboxylase (*gad)*, siderophore receptor (*fyuA)*, high molecular weight protein 2 non-ribosomal peptide synthetase (*irp2)*, iron transport protein (*sitA)* and Ferric aerobactin receptor (*iutA)*. Phylogroups B2, D, and F carried more variety of virulence genes including *air*, *terC*, *yfcv*, *gad*, *pic*, *senB*, *kpsE*, *OMPT*, *paC*, *papaF43 and iha* than the rest of the phylogroups. These findings are consistent with similar studies that have characterized these strains [10, 67–70]. The predominant occurrence of different virulent factors within phylogroup B2, F, and D may directly relate to their evolutionary fitness to establish and maintain themselves as well as cause infection. Similar virulence strains are shared and have been reported in animals potentiating the possibility of transmission to humans [71]. Like AMR, some of the virulence genes are carried on plasmids that further enhance their transmission [72–74].

## Conclusion

Our study confirmed the occurrence of the globally disseminated high-risk extra-intestinal *E*. *coli* pandemic clones exhibiting resistance to some of the critically important antibiotics and are a threat to the management and treatment of serious infections caused by multidrug-resistant (MDR) bacteria. We demonstrated the high genetic diversity of the isolates with multiple sequence types, serotypes, and fimbrial antigenic types distributed across 7 of the 8 *E*. *coli* phylogroups that possess broad resistome and virulome that enhances their transmission within the healthcare and community settings. The finding of third and fourth generation cephalosporins resistance genes against broad-spectrum β-lactam antimicrobial agents classified as critically important for human and animal medicine is of great concern. Some of the high-risk clones detected in this study have been reported in food, animals, and the environment highlighting the one-health nature of this problem that needs concerted efforts including long term genomic surveillance in all niches to generate the needed evidence to inform optimal containment strategies.

## Supporting information

S1 Fig Relative abundance of virulent genes among *E*. *coli* phylogroups. (TIF)

S1 File Phenotypic and genotypic characteristics of the isolates analyzed in this study. (XLSX)

## References

[1] Kocsis B, Gulyás D, Szabó D. Emergence and Dissemination of Extraintestinal Pathogenic High-Risk International Clones of Escherichia coli. Vol. 12, Life. MDPI; 2022. doi: 10.3390/life12122077 36556442 PMC9780897

[2] Manges AR, Geum HM, Guo A, Edens TJ, Fibke CD, Pitout JDD. Global extraintestinal pathogenic escherichia coli (Expec) lineages. Clin Microbiol Rev. 2019 Jul 1;32(3). doi: 10.1128/CMR.00135-18 31189557 PMC6589867

[3] Riley LW. Pandemic lineages of extraintestinal pathogenic Escherichia coli. Vol. 20, Clinical Microbiology and Infection. Blackwell Publishing Ltd; 2014. p. 380–90. 24766445 10.1111/1469-0691.12646

[4] Irenge LM, Ambroise J, Bearzatto B, Durant JF, Chirimwami RB, Gala JL. Whole-genome sequences of multidrug-resistant Escherichia coli in South-Kivu Province, Democratic Republic of Congo: Characterization of phylogenomic changes, virulence and resistance genes. BMC Infect Dis. 2019 Feb 11;19(1). doi: 10.1186/s12879-019-3763-3 30744567 PMC6371417

[5] Tegha G, Ciccone EJ, Krysiak R, Kaphatika J, Chikaonda T, Ndhlovu I, et al. Genomic epidemiology of escherichia coli isolates from a tertiary referral center in lilongwe, Malawi. Microb Genom. 2021;7(1):1–12. doi: 10.1099/mgen.0.000490 33295867 PMC8115906

[6] Eger E, Heiden SE, Korolew K, Bayingana C, Ndoli JM, Sendegeya A, et al. Circulation of Extended-Spectrum Beta-Lactamase-Producing Escherichia coli of Pandemic Sequence Types 131, 648, and 410 Among Hospitalized Patients, Caregivers, and the Community in Rwanda. Front Microbiol. 2021 May 14;12. doi: 10.3389/fmicb.2021.662575 34054764 PMC8160302

[7] Iramiot JS, Kajumbula H, Bazira J, De Villiers EP, Asiimwe BB. Whole genome sequences of multi-drug resistant Escherichia coli isolated in a Pastoralist Community of Western Uganda: Phylogenomic changes, virulence and resistant genes. PLoS One. 2020 May 1;15(5). 10.1371/journal.pone.0231852 PMC7259651 32469885

[8] Mathers AJ, Peirano G, Pitout JDD. The role of epidemic resistance plasmids and international high- risk clones in the spread of multidrug-resistant Enterobacteriaceae. Clin Microbiol Rev. 2015;28(3):565–91. doi: 10.1128/CMR.00116-14 25926236 PMC4405625

[9] Johnson JR, Tchesnokova V, Johnston B, Clabots C, Roberts PL, Billig M, et al. Abrupt emergence of a single dominant multidrug-resistant strain of Escherichia coli. Journal of Infectious Diseases. 2013;207(6):919–28. doi: 10.1093/infdis/jis933 23288927 PMC3571447

[10] Sora VM, Meroni G, Martino PA, Soggiu A, Bonizzi L, Zecconi A. Extraintestinal pathogenic escherichia coli: Virulence factors and antibiotic resistance. Pathogens. 2021 Nov 1;10(11). doi: 10.3390/pathogens10111355 34832511 PMC8618662

[11] Kudinha T, Johnson JR, Andrew SD, Kong F, Anderson P, Gilbert GL. Escherichia coli sequence type 131 as a prominent cause of antibiotic resistance among urinary Escherichia coli isolates from reproductive-age women. J Clin Microbiol. 2013 Oct;51(10):3270–6. doi: 10.1128/JCM.01315-13 23885001 PMC3811657

[12] Schaufler K, Semmler T, Wieler LH, Trott DJ, Pitout J, Peirano G, et al. Genomic and functional analysis of emerging virulent and multidrug-resistant Escherichia coli lineage sequence type 648. Antimicrob Agents Chemother. 2019 Jun 1;63(6). doi: 10.1128/AAC.00243-19 30885899 PMC6535536

[13] Ewers C, Bethe A, Stamm I, Grobbel M, Kopp PA, Guerra B, et al. CTX-M-15-D-ST648 escherichia coli from companion animals and horses: Another pandemic clone combining multiresistance and extraintestinal virulence? Journal of Antimicrobial Chemotherapy. 2014;69(5):1224–30. doi: 10.1093/jac/dkt516 24398338

[14] Hounmanou YMG, Wanyana A, Alafi S, Wabwire-Mangen F, Christensen H, Olsen JE, et al. Whole strains vs MGEs in short and longterm transmission of ESBL genes between healthcare and community settings in Uganda. Sci Rep. 2023 Dec 1;13(1). 10.1038/s41598-023-35879-x PMC10290109 37353515

[15] CLSI. Performance Standards for Antimicrobial Susceptibility Testing. CLSI supplement M100. Clinical and Laboratory Standards Institute. 33rd ed. 2023.

[16] Magiorakos AP, Srinivasan A, Carey RB, Carmeli Y, Falagas ME, Giske CG, et al. Multidrug-resistant, extensively drug-resistant and pandrug-resistant bacteria: An international expert proposal for interim standard definitions for acquired resistance. Clinical Microbiology and Infection. 2012;18(3):268–81. doi: 10.1111/j.1469-0691.2011.03570.x 21793988

[17] McGann P, Bunin JL, Snesrud E, Singh S, Maybank R, Ong AC, et al. Real time application of whole genome sequencing for outbreak investigation–What is an achievable turnaround time? Diagn Microbiol Infect Dis. 2016 Jul 1;85(3):277–82. doi: 10.1016/j.diagmicrobio.2016.04.020 27185645

[18] Kong Y. Btrim: A fast, lightweight adapter and quality trimming program for next-generation sequencing technologies. Genomics. 2011 Aug;98(2):152–3. doi: 10.1016/j.ygeno.2011.05.009 21651976

[19] Nederbragt AJ. On the middle ground between open source and commercial software—the case of the Newbler program. Vol. 15, Genome Biology. BioMed Central Ltd.; 2014. doi: 10.1186/gb4173 25180324 PMC4054848

[20] Tanizawa Y, Fujisawa T, Kaminuma E, Nakamura Y, Arita M. DFAST and DAGA: web-based integrated genome annotation tools and resources. Bioscience of Microbiota, Food and Health [Internet]. 2016;35(4):173–84. Available from: http://www.bacterio.net/lactobacillaceae. doi: 10.12938/bmfh.16-003 27867804 PMC5107635

[21] Kaas RS, Leekitcharoenphon P, Aarestrup FM, Lund O. Solving the problem of comparing whole bacterial genomes across different sequencing platforms. PLoS One. 2014 Aug 11;9(8). doi: 10.1371/journal.pone.0104984 25110940 PMC4128722

[22] Beghain J, Bridier-Nahmias A, Nagard H le, Denamur E, Clermont O. ClermonTyping: An easy-to-use and accurate in silico method for Escherichia genus strain phylotyping. Microb Genom. 2018 Jul 1;4 (7). doi: 10.1099/mgen.0.000192 29916797 PMC6113867

[23] Larsen MV., Cosentino S, Rasmussen S, Friis C, Hasman H, Marvig RL, et al. Multilocus sequence typing of total-genome-sequenced bacteria. J Clin Microbiol. 2012 Apr;50(4):1355–61. doi: 10.1128/JCM.06094-11 22238442 PMC3318499

[24] Joensen KG, Tetzschner AMM, Iguchi A, Aarestrup FM, Scheutz F. Rapid and easy in silico serotyping of Escherichia coli isolates by use of whole-genome sequencing data. J Clin Microbiol. 2015 Aug 1;53(8):2410–26. doi: 10.1128/JCM.00008-15 25972421 PMC4508402

[25] Roer L, Tchesnokova V, Allesøe R, Muradova M, Chattopadhyay S, Ahrenfeldt J, et al. Development of a Web Tool for Escherichia coli Subtyping Based on fimH Alleles [Internet]. 2017. Available from: https://journals.asm.org/journal/jcm 10.1128/JCM.00737-17 PMC5527432 28592545

[26] Price LB, Johnson JR, Aziz M, Clabots C, Johnston B, Tchesnokova V, et al. The epidemic of extended-spectrum-β-lactamase-producing Escherichia coli ST131 is driven by a single highly pathogenic subclone, H30-Rx. mBio. 2013 Dec 17;4(6). 10.1128/mBio.00377-13 PMC3870262 24345742

[27] Alcock BP, Huynh W, Chalil R, Smith KW, Raphenya AR, Wlodarski MA, et al. CARD 2023: expanded curation, support for machine learning, and resistome prediction at the Comprehensive Antibiotic Resistance Database. Nucleic Acids Res. 2023 Jan 6;51(1 D):D690–9. 36263822 10.1093/nar/gkac920 PMC9825576

[28] Feldgarden M, Brover V, Haft DH, Prasad AB, Slotta DJ, Tolstoy I, et al. Validating the AMRFINder tool and resistance gene database by using antimicrobial resistance genotype-phenotype correlations in a collection of isolates. Antimicrob Agents Chemother. 2019;63(11). 10.1128/AAC.00483-19 PMC6811410 31427293

[29] Bortolaia V, Kaas RS, Ruppe E, Roberts MC, Schwarz S, Cattoir V, et al. ResFinder 4.0 for predictions of phenotypes from genotypes. Journal of Antimicrobial Chemotherapy. 2020 Dec 1;75(12):3491–500. doi: 10.1093/jac/dkaa345 32780112 PMC7662176

[30] Joensen KG, Scheutz F, Lund O, Hasman H, Kaas RS, Nielsen EM, et al. Real-time whole-genome sequencing for routine typing, surveillance, and outbreak detection of verotoxigenic Escherichia coli. J Clin Microbiol. 2014;52(5):1501–10. doi: 10.1128/JCM.03617-13 24574290 PMC3993690

[31] Spurbeck RR, Dinh PC, Walk ST, Stapleton AE, Hooton TM, Nolan LK, et al. Escherichia coli isolates that carry vat, fyua, chua, and yfcv efficiently colonize the urinary tract. Infect Immun. 2012;80(12):4115–22. doi: 10.1128/IAI.00752-12 22966046 PMC3497434

[32] Carattoli A, Zankari E, Garciá-Fernández A, Larsen MV, Lund O, Villa L, et al. In Silico detection and typing of plasmids using plasmidfinder and plasmid multilocus sequence typing. Antimicrob Agents Chemother. 2014;58(7):3895–903. doi: 10.1128/AAC.02412-14 24777092 PMC4068535

[33] Arredondo-Alonso S, Rogers MRC, Braat JC, Verschuuren TD, Top J, Corander J, et al. mlplasmids: a user-friendly tool to predict plasmid-and chromosome-derived sequences for single species DATA SUMMARY. Available from: https://sarredondo.shinyapps. 10.1099/mgen.0.000224 PMC6321875 30383524

[34] Gilchrist CLM, Chooi YH. Clinker & clustermap.js: Automatic generation of gene cluster comparison figures. Bioinformatics. 2021 Aug 15;37(16):2473–5. 33459763 10.1093/bioinformatics/btab007

[35] Fernandes MR, Sellera FP, Moura Q, Gaspar VC, Cerdeira L, Lincopan N. International high-risk clonal lineages of CTX-M-producing Escherichia coli F-ST648 in free-roaming cats, South America. Infection, Genetics and Evolution. 2018 Dec 1;66:48–51. doi: 10.1016/j.meegid.2018.09.009 30227226

[36] Johnson TJ, Elnekave E, Miller EA, Munoz-Aguayo J, Figueroa CF, Johnston B, et al. Phylogenomic Analysis of Extraintestinal Pathogenic Escherichia coli Sequence Type 1193, an Emerging Multidrug-Resistant Clonal Group. Antimicrob Agents Chemother. 2019 Jan 1;63(1). doi: 10.1128/AAC.01913-18 30348668 PMC6325179

[37] Merino I, Hernández-García M, Turrientes MC, Pérez-Viso B, López-Fresneña N, Diaz-Agero C, et al. Emergence of ESBL-producing Escherichia coli ST131-C1-M27 clade colonizing patients in Europe. Journal of Antimicrobial Chemotherapy. 2018 Nov 1;73(11):2973–80. doi: 10.1093/jac/dky296 30124851

[38] Katale BZ, Misinzo G, Mshana SE, Chiyangi H, Campino S, Clark TG, et al. Genetic diversity and risk factors for the transmission of antimicrobial resistance across human, animals and environmental compartments in East Africa: A review. Vol. 9, Antimicrobial Resistance and Infection Control. BioMed Central Ltd; 2020. doi: 10.1186/s13756-020-00786-7 32762743 PMC7409632

[39] Murray CJ, Ikuta KS, Sharara F, Swetschinski L, Robles Aguilar G, Gray A, et al. Global burden of bacterial antimicrobial resistance in 2019: a systematic analysis. The Lancet. 2022 Feb 12;399(10325):629–55. 10.1016/S0140-6736(21)02724-0 PMC8841637 35065702

[40] Katongole P, Bulwadda Kisawuzi D, Kyobe Bbosa H, Patrick Kateete D, Florence Najjuka C. Phylogenetic groups and antimicrobial susceptibility patterns of uropathogenic Escherichia coli clinical isolates from patients at Mulago National Referral Hospital, Kampala, Uganda. F1000Res. 2019 Oct 30;8:1828.

[41] Alfinete NW, Bolukaoto JY, Heine L, Potgieter N, Barnard TG. Virulence and phylogenetic analysis of enteric pathogenic Escherichia coli isolated from children with diarrhoea in South Africa. International Journal of Infectious Diseases. 2022 Jan 1;114:226–32. doi: 10.1016/j.ijid.2021.11.017 34775113

[42] Vigil KJ, Johnson JR, Johnston BD, Kontoyiannis DP, Mulanovich VE, Raad II, et al. Escherichia coli pyomyositis: An emerging infectious disease among patients with hematologic malignancies. Clinical Infectious Diseases. 2010 Feb 1;50(3):374–80. doi: 10.1086/649866 20038242

[43] Ender PT, Gajanana D, Johnston B, Clabots C, Tamarkin FJ, Johnson JR. Transmission of an extended-spectrum-beta-lactamase-producing Escherichia coli (sequence type ST131) strain between a father and daughter resulting in septic shock and emphysematous pyelonephritis. J Clin Microbiol. 2009 Nov;47(11):3780–2. doi: 10.1128/JCM.01361-09 19741070 PMC2772609

[44] Nicolas-Chanoine MH, Bertrand X, Madec JY. Escherichia coli st131, an intriguing clonal group. Clin Microbiol Rev. 2014;27(3):543–74. doi: 10.1128/CMR.00125-13 24982321 PMC4135899

[45] Mandomando I, Vubil D, Boisen N, Quintó L, Ruiz J, Sigaúque B, et al. Escherichia coli st131 clones harbouring AGGR and AAF/V fimbriae causing bacteremia in Mozambican children: Emergence of new variant of FIMH27 subclone. PLoS Negl Trop Dis. 2020 May 1;14(5):1–21. doi: 10.1371/journal.pntd.0008274 32357189 PMC7219792

[46] Zhong YM, Liu WE, Meng Q, Li Y. Escherichia coli O25B-ST131 and O16-ST131 causing urinary tract infection in women in Changsha, China: Molecular epidemiology and clinical characteristics. Infect Drug Resist. 2019;12:2693–702. doi: 10.2147/IDR.S212658 31564918 PMC6722436

[47] Dahbi G, Mora A, López C, Alonso MP, Mamani R, Marzoa J, et al. Emergence of new variants of ST131 clonal group among extraintestinal pathogenic Escherichia coli producing extended-spectrum β-lactamases. Int J Antimicrob Agents. 2013 Oct;42(4):347–51. 23992646 10.1016/j.ijantimicag.2013.06.017

[48] Blanc V, Leflon-Guibout V, Blanco J, Haenni M, Madec JY, Rafignon G, et al. Prevalence of day-care centre children (France) with faecal CTX-M-producing Escherichia coli comprising O25b: H4 and O16: H5 ST131 strains. Journal of Antimicrobial Chemotherapy. 2014;69(5):1231–7. doi: 10.1093/jac/dkt519 24402502

[49] Decano AG, Pettigrew K, Sabiiti W, Sloan DJ, Neema S, Bazira J, et al. Pan-resistome characterization of uropathogenic escherichia coli and klebsiella pneumoniae strains circulating in uganda and kenya, isolated from 2017–2018. Antibiotics. 2021 Dec 1;10(12). doi: 10.3390/antibiotics10121547 34943759 PMC8698711

[50] Colpan A, Johnston B, Porter S, Clabots C, Anway R, Thao L, et al. Escherichia coli sequence type 131 (ST131) subclone h30 as an emergent multidrug-resistant pathogen among US Veterans. Clinical Infectious Diseases. 2013 Nov 1;57(9):1256–65. doi: 10.1093/cid/cit503 23926176 PMC3792724

[51] Banerjee R, Robicsek A, Kuskowski MA, Porter S, Johnston BD, Sokurenko E, et al. Molecular epidemiology of Escherichia coli sequence type 131 and its H30 and H30-Rx subclones among extended-spectrum-β-lactamase-positive and -negative E. coli clinical isolates from the Chicago region, 2007 to 2010. Antimicrob Agents Chemother. 2013 Dec;57(12):6385–8. 24080662 10.1128/AAC.01604-13 PMC3837873

[52] Blanco J, Mora A, Mamani R, López C, Blanco M, Dahbi G, et al. Four main virotypes among extended-spectrum-β-lactamase-producing isolates of Escherichia coli O25b:H4-B2-ST131: Bacterial, epidemiological, and clinical characteristics. J Clin Microbiol. 2013 Oct;51(10):3358–67. 23926164 10.1128/JCM.01555-13 PMC3811668

[53] Rogers BA, Sidjabat HE, Paterson DL. Escherichia coli O25b-ST131: A pandemic, multiresistant, community-associated strain. Journal of Antimicrobial Chemotherapy. 2011 Jan;66(1):1–14. doi: 10.1093/jac/dkq415 21081548

[54] Matsumura Y, Pitout JDD, Gomi R, Matsuda T, Noguchi T, Yamamoto M, et al. Global Escherichia coli sequence type 131 clade with blaCTX-M-27 gene. Emerg Infect Dis. 2016 Nov 1;22(11):1900–7. doi: 10.3201/eid2211.160519 27767006 PMC5088012

[55] Sherchan JB, Hayakawa K, Miyoshi-Akiyama T, Ohmagari N, Kirikae T, Nagamatsu M, et al. Clinical epidemiology and molecular analysis of extended-spectrum-β-lactamase-producing Escherichia coli in Nepal: Characteristics of sequence types 131 and 648. Antimicrob Agents Chemother. 2015 Jun 1;59(6):3424–32. 25824221 10.1128/AAC.00270-15 PMC4432170

[56] Ampaire L, Muhindo A, Orikiriza P, Mwanga-Amumpaire J, Bebell L, Boum Y. A review of antimicrobial resistance in East Africa. Vol. 5, African Journal of Laboratory Medicine. AOSIS OpenJournals Publishing AOSIS (Pty) Ltd; 2016. doi: 10.4102/ajlm.v5i1.432 28879114 PMC5436405

[57] Mboowa G, Aruhomukama D, Sserwadda I, Kitutu FE, Davtyan H, Owiti P, et al. Increasing antimicrobial resistance in surgical wards at mulago national referral hospital, uganda, from 2014 to 2018—cause for concern? Trop Med Infect Dis. 2021 Jun 1;6(2). doi: 10.3390/tropicalmed6020082 34069345 PMC8163195

[58] Seni J, Najjuka CF, Kateete DP, Makobore P, Joloba ML, Kajumbula H, et al. Antimicrobial resistance in hospitalized surgical patients: A silently emerging public health concern in Uganda. BMC Res Notes. 2013;6(1). doi: 10.1186/1756-0500-6-298 23890206 PMC3729663

[59] Oteo J, Diestra K, Juan C, Bautista V, Novais Â, Pérez-Vázquez M, et al. Extended-spectrum β-lactamase-producing Escherichia coli in Spain belong to a large variety of multilocus sequence typing types, including ST10 complex/A, ST23 complex/A and ST131/B2. Int J Antimicrob Agents. 2009 Aug;34(2):173–6. 19464856 10.1016/j.ijantimicag.2009.03.006

[60] Matsumura Y, Johnson JR, Yamamoto M, Nagao M, Tanaka M, Takakura S, et al. CTX-M-27- and CTX-M-14-producing, ciprofloxacin-resistant Escherichia coli of the H30 subclonal group within ST131 drive a Japanese regional ESBL epidemic. Journal of Antimicrobial Chemotherapy. 2014 Nov 12;70(6):1639–49. 10.1093/jac/dkv017 25687644

[61] Rohde AM, Zweigner J, Wiese-Posselt M, Schwab F, Behnke M, Kola A, et al. Prevalence of third-generation cephalosporin-resistant Enterobacterales colonization on hospital admission and ESBL genotype-specific risk factors: A cross-sectional study in six German university hospitals. Journal of Antimicrobial Chemotherapy. 2020;75(6):1631–8. doi: 10.1093/jac/dkaa052 32173738

[62] Flament-Simon SC, García V, Duprilot M, Mayer N, Alonso MP, García-Meniño I, et al. High Prevalence of ST131 Subclades C2-H30Rx and C1-M27 Among Extended-Spectrum β-Lactamase-Producing Escherichia coli Causing Human Extraintestinal Infections in Patients From Two Hospitals of Spain and France During 2015. Front Cell Infect Microbiol. 2020 Mar 24;10. 10.3389/fcimb.2020.00125 PMC7105571 32266173

[63] Wachino JI, Yamane K, Suzuki S, Kimura K, Arakawa Y. Prevalence of fosfomycin resistance among CTX-M-producing Escherichia coli clinical isolates in Japan and identification of novel plasmid-mediated fosfomycin-modifying enzymes. Antimicrob Agents Chemother. 2010;54(7):3061–4. doi: 10.1128/AAC.01834-09 20404116 PMC2897269

[64] Hou J, Huang X, Deng Y, He L, Yang T, Zeng Z, et al. Dissemination of the fosfomycin resistance gene fosA3 with CTX-M β-lactamase genes and rmtB carried on incfII plasmids among escherichia coli isolates from pets in China. Antimicrob Agents Chemother. 2012 Apr;56(4):2135–8. 22232290 10.1128/AAC.05104-11 PMC3318358

[65] Falagas ME, Kastoris AC, Kapaskelis AM, Karageorgopoulos DE. Fosfomycin for the treatment of multidrug-resistant, including extended-spectrum β-lactamase producing, Enterobacteriaceae infections: a systematic review. Vol. 10, The Lancet Infectious Diseases. 2010. p. 43–50. 20129148 10.1016/S1473-3099(09)70325-1

[66] Zhang X, Ma M, Cheng Y, Huang Y, Tan Y, Yang Y, et al. Spread and Molecular Characteristics of Enterobacteriaceae Carrying fosA -Like Genes from Farms in China. Microbiol Spectr. 2022 Aug 31;10(4). doi: 10.1128/spectrum.00545-22 35852324 PMC9431306

[67] Terlizzi ME, Gribaudo G, Maffei ME. UroPathogenic Escherichia coli (UPEC) infections: Virulence factors, bladder responses, antibiotic, and non-antibiotic antimicrobial strategies. Vol. 8, Frontiers in Microbiology. Frontiers Media S.A.; 2017. doi: 10.3389/fmicb.2017.01566 28861072 PMC5559502

[68] Ballesteros-Monrreal MG, Arenas-Hernández MMP, Enciso-Martínez Y, Martínez-De la Peña CF, Rocha-Gracia RDC, Lozano-Zaraín P, et al. Virulence and resistance determinants of uropathogenic escherichia coli strains isolated from pregnant and non-pregnant women from two states in Mexico. Infect Drug Resist. 2020;13:295–310. doi: 10.2147/IDR.S226215 32099421 PMC6997036

[69] Klemm P, Hancock V, Schembri MA. Fimbrial adhesins from extraintestinal Escherichia coli. Vol. 2, Environmental Microbiology Reports. 2010. p. 628–40. doi: 10.1111/j.1758-2229.2010.00166.x 23766248

[70] Tarchouna M, Ferjani A, Ben-Selma W, Boukadida J. Distribution of uropathogenic virulence genes in Escherichia coli isolated from patients with urinary tract infection. International Journal of Infectious Diseases. 2013 Jun;17(6). doi: 10.1016/j.ijid.2013.01.025 23510539

[71] Obala T, Arojjo SO, Afayoa M, Ikwap K, Erume J. The role of Escherichia coli in the etiology of piglet diarrhea in selected pig producing districts of central Uganda. African Journal of Clinical and Experimental Microbiology. 2021 Sep 27;22(4):515–25.

[72] Tivendale KA, Logue CM, Kariyawasam S, Jordan D, Hussein A, Li G, et al. Avian-pathogenic Escherichia coli strains are similar to neonatal meningitis E. coli strains and are able to cause meningitis in the rat model of human disease. Infect Immun. 2010 Aug;78(8):3412–9. doi: 10.1128/IAI.00347-10 20515929 PMC2916289

[73] Rodriguez-Siek KE, Giddings CW, Doetkott C, Johnson TJ, Fakhr MK, Nolan LK. Comparison of Escherichia coli isolates implicated in human urinary tract infection and avian colibacillosis. Microbiology (N Y). 2005 Jun;151(6):2097–110. doi: 10.1099/mic.0.27499-0 15942016

[74] Johnson JR, Kuskowski MA, O’Bryan TT, Colodner R, Raz R. Virulence genotype and phylogenetic origin in relation to antibiotic resistance profile among Escherichia coli urine sample isolates from Israeli women with acute uncomplicated cystitis. Vol. 49, Antimicrobial Agents and Chemotherapy. 2005. p. 26–31. doi: 10.1128/AAC.49.1.26-31.2005 15616271 PMC538882

