# Supplementary figures and images for: Resistome and virulome of high-risk pandemic clones of multidrug-resistant extra-intestinal pathogenic Escherichia coli (ExPEC) isolated from tertiary healthcare settings in Uganda
Source: PLoS One. 2023 Nov 22;18(11):e0294424. doi: 10.1371/journal.pone.0294424 (PMC10664879; doi:10.1371/journal.pone.0294424)

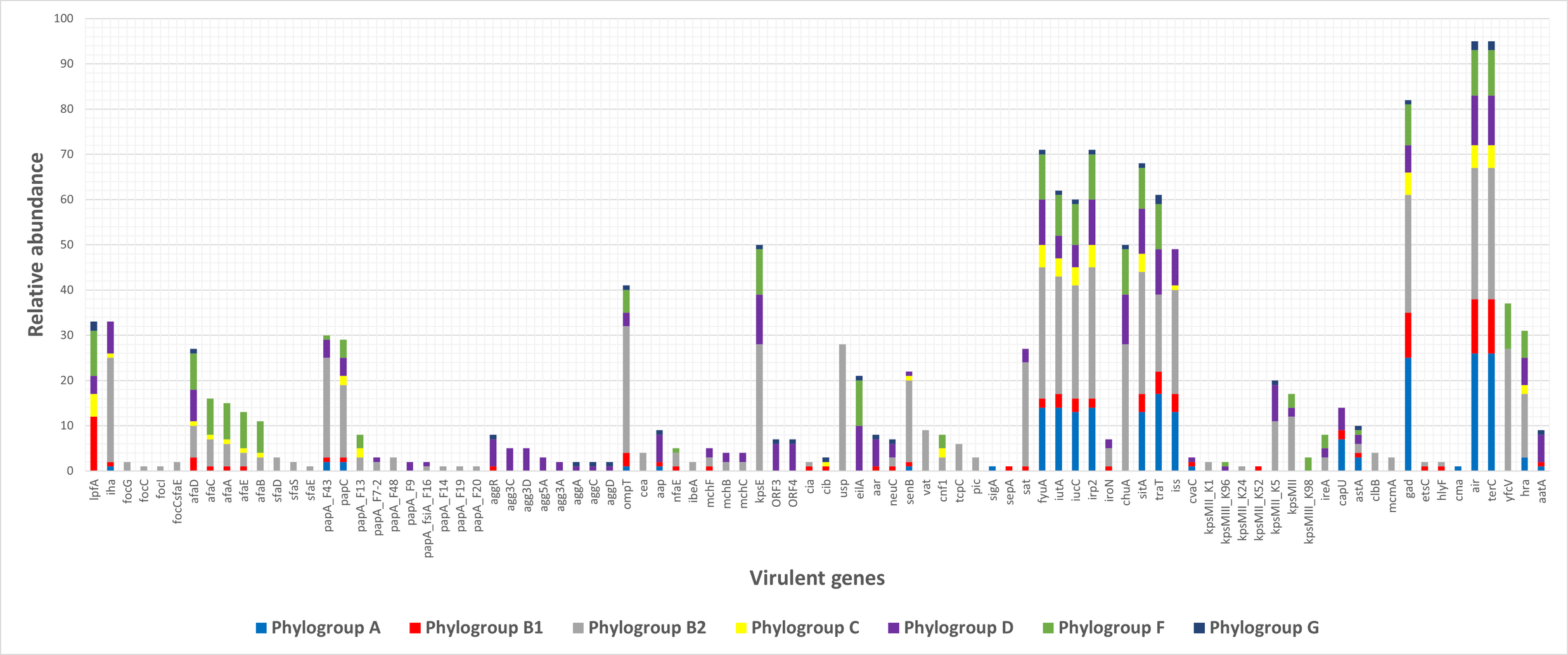

Supplement: S1 Fig — (TIF) [file pone.0294424.s001.tif]
